# Renal Transplantation in Patients With Urinary Diversion—Bypassing the Conduit: A Case Report

**DOI:** 10.1155/crit/6764308

**Published:** 2025-02-17

**Authors:** Matthew D. Wainstein, Obi Ekwenna

**Affiliations:** ^1^College of Medicine and Life Sciences, University of Toledo, Toledo, Ohio, USA; ^2^Department of Urology and Transplantation, College of Medicine and Life Sciences, University of Toledo, Toledo, Ohio, USA

**Keywords:** ileal conduit, renal transplant, ureteral diversion, uretero-uretero anastomosis

## Abstract

Kidney transplantation in patients with supravesical urinary diversions is a relatively rare procedure. The typical approach for establishing urinary drainage in these patients is through an ureteroileal anastomosis. However, a tension-free ureteroileal anastomosis can be difficult to achieve based on variations in the anatomy of the donor ureter or recipient conduit. An alternative technique can be to create an anastomosis between the donor and recipient ureter, although reports of this technique in the last 20 years have been sparse. Here, we present two cases of patients with prior ileal conduits who underwent successful renal transplantation with uretero-uretero anastomoses.

## 1. Introduction

Congenital and acquired urinary anomalies can contribute to end-stage renal disease (ESRD), requiring kidney transplantation with urine drainage into a urinary conduit [1]. Kidney transplantation into patients with supravesical urinary diversions is a relatively rare procedure, with single-center studies reporting frequencies between 0.4% and 2.3% [1, 2]. While there has been apprehension to perform transplants on these patients due to fear of graft rejection and infection, reports in the last 25 years have shown comparable outcomes to renal transplantation in normal patients [1–4]. The typical approach for establishing urinary drainage in these patients is through a ureteroileal anastomosis [1–3]. Creating a uretero-uretero anastomosis can be an alternative approach in instances where either the donor ureter or the recipient conduit does not allow for a tension-free ureteroileal anastomosis [4]. A few reports have described utilization of this technique with good results aside from a higher incidence of urinary tract infection (UTI) [4, 5]. Here, we present two cases of patients with prior ileal conduits who underwent successful renal transplantation with uretero-uretero anastomoses.

## 2. Case Presentation

### 2.1. Case 1

A 64-year-old African American male was referred to our clinic for evaluation of kidney transplantation in November of 2019. The patient had a history of ESRD of multifactorial origin, including having a urostomy with urinary diversion since the age of 6. He had been on maintenance dialysis for about 9 years.

**Figure 1:**
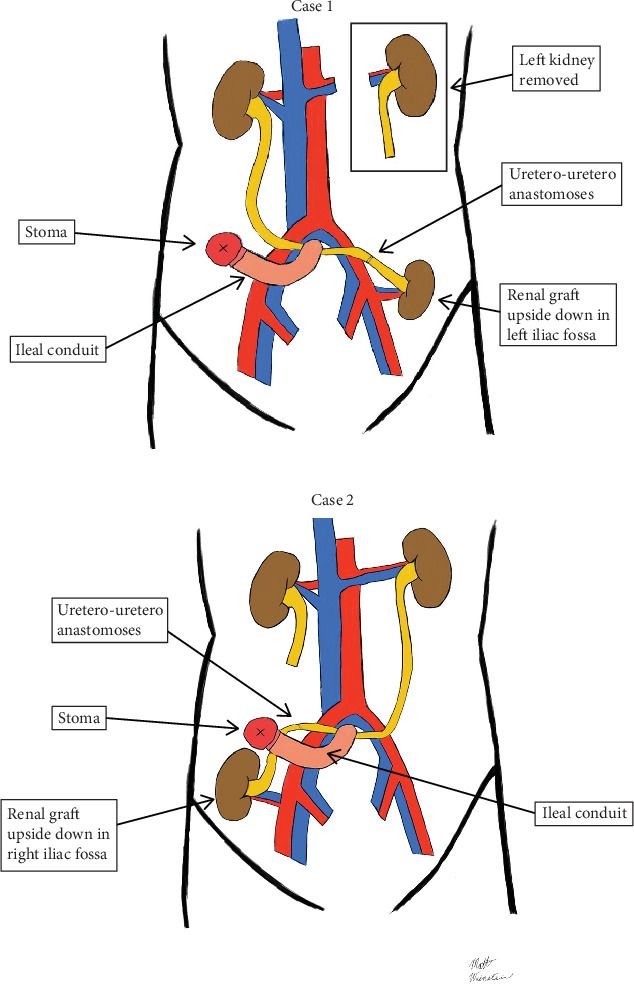
(a, b) Uretero-uretero anastomoses between native ureter and donor ureter in kidney transplantation with pre-existing ileal conduit.

The patient underwent a deceased donor renal transplant 21 months ago. He received a left kidney allograft from a donation after cardiac death (DCD) donor with a kidney donor profile index (KDPI) of 22%. The right lower quadrant was chosen for implantation due to the patient's ileal conduit being on the left side. The extraperitoneal space was accessed using a modified Gibson incision. After exposing the external iliac vessels, the renal vessels were anastomosed in an end-to-side fashion. The right native ureter was then ligated and anastomosed with the donor ureter end to end. The patient's native kidney was kept in situ, and no ureteral stenting was placed (Figure 1(a)). The operation lasted approximately 5.5 h, and the patient tolerated the procedure well with no intra- or postoperative complications.

Twenty months posttransplant, the patient has not experienced any rejection. He had one episode of UTI which resolved with oral antibiotics. The patient's serum creatinine at 18 months posttransplant was 1.52.

### 2.2. Case 2

A 68-year-old male with a history of ESRD secondary to chronic reflux nephropathy and bladder exstrophy with ileal conduit urinary diversion at age 15 was referred to our clinic for evaluation. He was maintained on dialysis for 21 months prior to transplant.

The patient underwent an uneventful left native nephrectomy and deceased donor transplant 16 months ago. The allograft was a right kidney from a 44-year-old donation after brain death (DBD) donor with a KDPI of 23%. The left lower quadrant was chosen for implantation due to the patient's ileal conduit being on the right side. A modified Gibson incision was created to access the extraperitoneal space. The external iliac vessels were then exposed and anastomosed with the renal vessels in an end-to-side fashion. The recipient's left ureter and the donor ureter were then identified and brought into the field. The ureters were then anastomosed in an end-to-end fashion (Figure 1(b)). No ureteral stenting was placed, and a Jackson–Pratt (JP) drain was positioned near the kidney prior to closure. In total, the operation lasted approximately 6.5 h. The patient tolerated the procedure well and experienced no intraoperative complications. He was started on immunosuppressive therapy and was discharged on post-op Day 4.

Sixteen months posttransplant, the patient continues to do well with excellent graft function. His most recent serum creatinine about 1 year posttransplant was 1.24.

## 3. Discussion

Patients with congenital abnormalities of the lower urinary tract often have associated comorbidities often leading to renal failure at a young age [1, 4]. While kidney transplantation into ileal conduits was first reported in 1966 [6], there has been reluctance to perform transplants in these patients due to fear of postsurgical infection and complications. However, numerous studies have demonstrated comparable postsurgical complication rates to transplants in normal patients [1–4]. While the native bladder is typically the best site for urinary drainage in kidney transplantation, establishing drainage into an intestinal conduit can be a valid option for patients with supravesical urinary diversions.

The standard technique when performing kidney transplantation in patients with ileal conduits involves implanting the donor ureter into the pre-existing ileal loop [1–3]. However, creating a tension-free ureteroileal anastomosis can be difficult to achieve in situations where either the donor ureter is short or the ileal conduit is contracted. In our case, the conduits were contracted making it impossible to identify during preoperative evaluation for transplant clearance. The alternative approach to create a ureteroureterostomy between the donor ureter and ipsilateral native ureter was made preoperatively. While the literature discussing this technique in the last 25 years has been sparse, few studies have shown that this can be a feasible option in select situations [4, 5]. Our results support this, as both patients who received renal transplants with our technique continue to have good-functioning allografts over 1 year posttransplant. While one patient did experience a recent UTI and an acute kidney injury following acute pneumonia, his renal function stabilized after treatment.

In order to prevent the development of ureteral strictures or other complications due to impaired ureteral blood supply, it is recommended that this technique should only be done in patients with well-vascularized distal ureters [4, 5]. Thus, in patients requiring ileal conduit formation before transplantation, adequate time should be allowed for the distal ureter to become revascularized [4]. Because both of our patients had long-standing ileal conduits prior to their transplants, this was not an issue for us.

While ureteroileal anastomosis is the preferred technique for renal transplant in patients with ileal conduits, uretero-uretero anastomosis is a valid alternative in select situations. Transplant patients should be aware of this technique and when it may or may not be indicated.

## Data Availability

Data sharing is not applicable to this article as no new data were created or analyzed in this study.

## Nomenclature

DBD: donation after brain death
DCD: donation after cardiac death
ESRD: end-stage renal disease
KDPI: kidney donor profile index
UTI: urinary tract infection
